# Choroid plexus volume associated with cognitive decline in rural older adults: A population‐based cohort study

**DOI:** 10.1002/alz70860_101796

**Published:** 2025-12-23

**Authors:** Jiafeng Wang, Xiaoyu Wang, Qianqian Xie, Yifei Ren, Chunyan Li, Xiaodong Han, Yan Wang, Ziwei Chen, Huisi Zhang, Tingting Hou, yongxiang wang, Lin Cong, Lin Song, Yifeng Du, Chengxuan Qiu

**Affiliations:** ^1^ Key Laboratory of Endocrine Glucose & Lipids Metabolism and Brain Aging, Ministry of Education; Department of Neurology, Shandong Provincial Hospital Affiliated to Shandong First Medical University, Jinan, Shandong, China; ^2^ Department of Neurology, Shandong Provincial Hospital, Shandong University, Jinan, Shandong, China; ^3^ Shandong Provincial Hospital Affiliated to Shandong First Medical University, Jinan, Shandong, China; ^4^ Innovation Center for Neurological Disorders and Department of Neurology, Xuanwu Hospital, Capital Medical University, National Clinical Research Center for Geriatric Diseases, Beijing, Beijing, China; ^5^ Department of Neurology, Shandong Provincial Hospital Affiliated to Shandong First Medical University, Jinan, Shandong, China; ^6^ Medical Science and Technology Innovation Center, Shandong First Medical University & Shandong Academy of Medical Sciences, Jinan, Shandong, China; ^7^ Department of Neurology, Shandong Provincial Hospital affiliated to Shandong First Medical University, Jinan, Shandong, China; ^8^ Institute of Brain Science and Brain‐Inspired Research, Shandong First Medical University & Shandong Academy of Medical Sciences, Jinan, Shandong, China

## Abstract

**Background:**

The choroid plexus (ChP) is a highly vascularized structure within the ventricles that produces cerebrospinal fluid (CSF) and forms the blood‐CSF barrier. We sought to investigate the longitudinal association of ChP volume with cognitive decline in older adults.

**Method:**

The population‐based cohort study enrolled 176 dementia‐free rural residents (age ≥60 years; 41.5% women) who underwent magnetic resonance imaging scans in 2015‐2016. ChP volume was automatically segmented using three‐dimensional T1‐weighted sequences. Cognitive function was assessed using the Mini‐Mental State Examination (MMSE) at baseline in 2014 and reassessed in 2018 and 2021. Data were analyzed using linear mixed‐effects and mediation models.

**Result:**

Compared with the medium tertile, both the lower and the upper tertiles of ChP volume were significantly associated the multivariable‐adjusted β coefficient of ‐0.26 (95% confidence interval ‐0.48 to ‐0.03) and ‐0.45 (‐0.68 to ‐0.21), respectively, for MMSE score decline; the observed associations were statistically evident mainly among men not in women (*p* for ChP volume × sex interaction < 0.05) and in participants aged ≥70 years not in those <70 years of age (*p* for ChP volume × age interaction < 0.01). The mediation analysis suggested that inferior lateral ventricular (ILV) volume and quantitative medial temporal lobe atrophy (QMTA) significantly mediated 21.4% and 20.5%, respectively, of the association of upper ChP volume with the MMSE score decline.

**Conclusion:**

This study reveals an inverted U‐shaped relationship between ChP volume and global cognitive decline, especially in men and young‐old adults (e.g., age <70 years), and further suggests that the association is partially mediated by the ILV volume and QMT.

